# Improving vaccination coverage and offering vaccine to all school-age children allowed uninterrupted in-person schooling in King County, WA: Modeling analysis

**DOI:** 10.3934/mbe.2022266

**Published:** 2022-04-01

**Authors:** Chloe Bracis, Mia Moore, David A. Swan, Laura Matrajt, Larissa Anderson, Daniel B. Reeves, Eileen Burns, Joshua T. Schiffer, Dobromir Dimitrov

**Affiliations:** 1 Université Grenoble Alpes, TIMC-IMAG/MAGE, Grenoble 38000, France; 2 Vaccine and Infectious Disease Division, Fred Hutchinson Cancer Research Center, Seattle, WA, USA; 3 Independent Researcher, Seattle, WA, USA; 4 Clinical Research Division, Fred Hutchinson Cancer Research Center; Seattle, WA, USA; 5 Department of Medicine, University of Washington, Seattle, WA, USA; 6 Department of Applied Mathematics, University of Washington, Seattle, WA, USA

**Keywords:** COVID-19 vaccination, mathematical modeling, variants of concern, epidemiology, age structured model

## Abstract

The rapid spread of highly transmissible SARS-CoV-2 variants combined with slowing pace of vaccination in Fall 2021 created uncertainty around the future trajectory of the epidemic in King County, Washington, USA. We analyzed the benefits of offering vaccination to children ages 5–11 and expanding the overall vaccination coverage using mathematical modeling. We adapted a mathematical model of SARS-CoV-2 transmission, calibrated to data from King County, Washington, to simulate scenarios of vaccinating children aged 5–11 with different starting dates and different proportions of physical interactions (PPI) in schools being restored. Dynamic social distancing was implemented in response to changes in weekly hospitalizations. Reduction of hospitalizations and estimated time under additional social distancing measures are reported over the 2021–2022 school year. In the scenario with 85% vaccination coverage of 12+ year-olds, offering early vaccination to children aged 5–11 with 75% PPI was predicted to prevent 756 (median, IQR 301–1434) hospitalizations cutting youth hospitalizations in half compared to no vaccination and largely reducing the need for additional social distancing measures over the school year. If, in addition, 90% overall vaccination coverage was reached, 60% of remaining hospitalizations would be averted and the need for increased social distancing would almost certainly be avoided. Our work suggests that uninterrupted in-person schooling in King County was partly possible because reasonable precaution measures were taken at schools to reduce infectious contacts. Rapid vaccination of all school-aged children provides meaningful reduction of the COVID-19 health burden over this school year but only if implemented early. It remains critical to vaccinate as many people as possible to limit the morbidity and mortality associated with future epidemic waves.

## 1. Introduction

Several effective SARS-CoV-2 vaccines were initially authorized for adults in late 2020 and later expanded to 12–16 year-old youths in May 2021, creating hope that the COVID-19 pandemic could be controlled and that social distancing and mask mandates could be permanently relaxed. With a rapidly increasing proportion of the population vaccinated in the United States, local and state authorities began loosening COVID-19 restrictions in spring 2021 and lifting all restrictions for vaccinated people during summer of 2021 in many jurisdictions [1].

Several highly transmissible variants of concern began to emerge. The B.1.1.7 (Alpha) variant quicky predominated in the first 3–4 months of the year but was soon replaced by the B.1.167.2 (Delta) variant over the summer across the globe [2,3]. Aside from being approximately 60% more transmissible than the Alpha variant, the Delta variant also had a greater proportional likelihood of hospitalization and need for intensive care (ICU) [4–6]. While vaccine effectiveness against different variants is still being evaluated, initial evidence suggested a decrease in protection against symptomatic infection after 6 months following vaccination [7,8]. In comparison, protection against severe disease and hospitalization remained relatively high against the Delta variant but may wane against the Omicron variant [9–14]. Together, the shifting dynamics created by ongoing vaccination, increased background immunity due to the surge of infections associated with the Delta variant, shifting characteristics of emerging viral variants and changes in social distancing and masking practices have created uncertainty around the future trajectory of epidemic in the U.S. and elsewhere.

A critical unknown question was how to safely open schools, which had remained closed across most of the U.S. during the 2020–2021 school year, and to estimate the impact of vaccinating children under the age of 12, particularly as clinical trials reported high vaccine effectiveness in children 5–11 [15] and the U.S. Food and Drug Administration (FDA) authorized the Pfizer-BioNTech COVID-19 vaccine for emergency use in this age group on October 29, 2021 [16].

King County, Washington coordinated a rapid vaccination rollout which resulted in more than 76% of the eligible (age 12 and older) population fully vaccinated by August 15, 2021 [17]. As a large proportion of the unvaccinated population at that time were school aged children, the question of the possible impact of reopening schools in Fall 2021 in the context of a surging Delta variant was of critical importance. Schools in the county were predominately closed since the beginning of the epidemic in March 2020 with most education being delivered online. Hybrid options of schooling were implemented throughout Washington state in Spring 2021 without evident impact on ongoing transmission [18]. All school districts in King County returned to full-time in-person instruction in September 2021, with only a small pilot online program for high-risk students. All districts developed guidelines to limit SARS-CoV-2 spread, which included the mandatory use of masks, adequate building ventilation, and other interventions to reduce contacts in schools below their pre-COVID-19 levels.

There has been extensive modeling work around vaccination and reopening strategies at different scales: from national level [19–23] to studies analyzing the impact of reopening schools and universities [24–27]. In an earlier analysis, conducted during the initial COVID-19 outbreak in Spring 2020, we concluded that school reopening would not significantly increase SARS-CoV-2 transmission if a well-coordinated combination of non-pharmaceutical mitigation measures were in place [28]. Other modeling studies suggested that infection rates through late November would exceed 75% and 90% among high school and elementary students respectively in the absence of masking and testing but that infection rates could be lowered significantly with both interventions [29].

In this study we used mathematical models to investigate the contribution that in-person schooling is expected to have on COVID-19 hospitalizations, under the current projections of vaccination coverage in King County and the expansion of the Delta variant in the area. We also analyzed the expected benefits of offering vaccination to children aged 5–11 starting on October 1, 2021 relative to: 1) delaying this access by another 3 months or 2) continuing to vaccinate individuals 12 years and older only. Finally, we studied how waning immunity of vaccine protection may affect these results and if it might change the recommendations regarding how schools should operate in King County.

## 2. Methods

**Figure 1.**
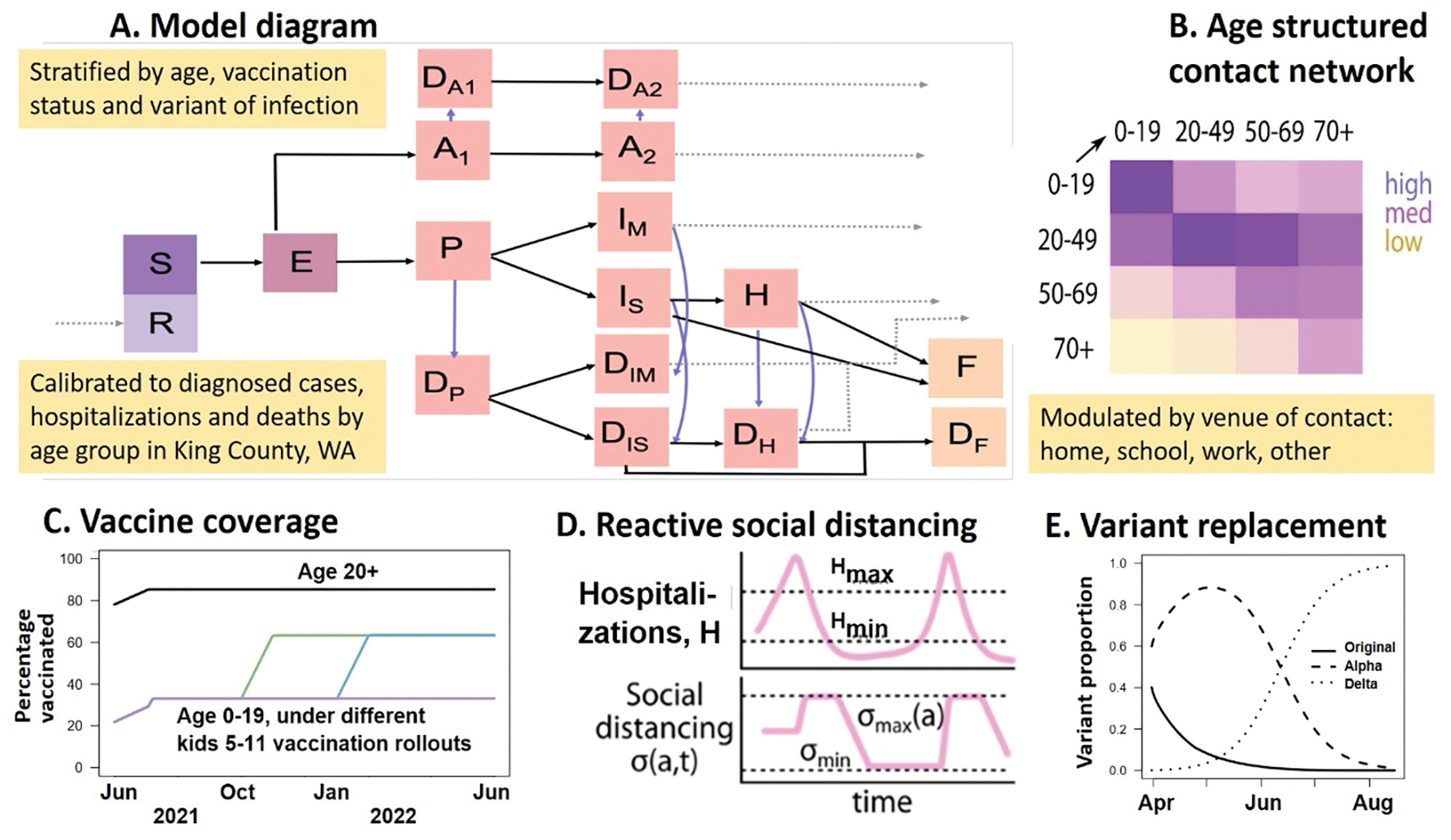
Modeling framework. A) Model diagram showing the progression of susceptible (S) individuals to exposed (E) who are not infectious, asymptomatic (A), pre-symptomatic (P), mild (I_M_) and severe (I_S_) symptomatic and hospitalized (H) with corresponding diagnosed states (D). All infections result in recovery (R) with partial immunity or fatality (F, D_F_), where fill color indicates susceptible (purple), infected and not contagious (dark pink), infected and contagious (light pink), and dead (yellow) with dashed gray lines indicating transition to the recovered state; B) Contact matrix; C) Vaccination coverage under different rollouts; D) Reactive social distancing in response to changes in hospitalization count; E) Variant replacement over time.

We adapted a deterministic compartment model, developed and iteratively improved by our team, which describes the epidemic dynamics in King County, Washington, USA [28,30–32]. Our model (Figure 1A and Figure S1) stratifies the population by age (0–19 years, 20–49 years, 50–69 years, and 70+ years), infection status (susceptible, exposed, asymptomatic, pre-symptomatic, symptomatic mild, symptomatic severe), clinical status (undiagnosed, diagnosed, hospitalized), immunity (susceptible, vaccinated, recovered from natural infection), and infecting strain (original, Alpha, Delta). The model was parameterized with larger infectivity early in the infectious period assuming that 44% of the infectivity is concentrated in the pre-symptomatic stage [33]. This has been highlighted as one of the key differences of SARS-CoV-2 infections compared to flu [34]. Detailed description of the model structure, equations and parameters is included in the Supplement.

Major changes to previous model versions, implemented in this study, include separate compartments for mild and severe disease, age-specific relative susceptibility strata, vaccine efficacy against hospitalization, ability to handle multiple variants with increased transmissibility and virulence as well as differential vaccine efficacies against each variant. We now stratify the contact matrix by venue (school, home, work, other) and can adjust local mixing parameters to implement reactive reduction in contacts at each venue (Table S3). We have no contact data specific to King County but used analysis representative for the United States [35]. We implemented reactive social distancing (SD) allowing numbers of cases and hospitalizations to fluctuate due to the community response to the epidemic [36]. We used intermediate range of contact reduction, more than pre-pandemic levels but less than a full lockdown [28,30–32].

We used Approximate Bayesian Computation [37] to select 100 parameterizations (see Figures S2, S3, S4 and S5) which minimized the distance between the normalized model outputs and normalized data for the 12 metrics (diagnosed cases, hospitalizations, and deaths for four age groups). Extended simulations beyond the calibration period up to Sept 1, 2021 were used for model validation (Figure S6).

All simulations included in this analysis started on June 1^s^, 2021 with initial conditions calculated based on estimates of cumulative incidence, variant prevalence and vaccination coverage by age group as reported in King County. See Supplement for a complete description of the model structure (Table S1), parameters (Table S2), initialization, and calibration.

### 2.1. Simulated scenarios

Our analysis is focused on the impact of vaccination programs based on: i) extending vaccination to children aged 5–11 with different starting dates (October 2021 and January 2022). It was implemented by expanding the coverage in the youngest group (0–19 year-old) based on the population fraction that 5–11 group represents; ii) different proportions of physical interactions (PPI) at schools restored varying from completely closed to fully opened. It is implemented by rescaling the school-related contact matrix to simulate different proportions of contacts in schools and iii) improving the overall vaccination coverage among the eligible population. The set of scenarios included in the main and additional analyses are listed in Table 2.

### 2.2. Outcomes of interest

We measured the impact of different vaccination programs by comparing the cumulative hospitalizations (overall, by vaccination status and by age group), peak hospitalizations (that is the maximum number of current hospitalizations), the proportion of simulations requiring additional mitigation measures and the proportion of time under elevated social distancing calculated over the school year (September 2021–May 2022). For reference, there are approximately 3900 inpatient hospital beds in King County [38]. To study the impact of early vaccination of children aged 5–11 we also estimated the percentage reduction of hospitalizations over the same period compared to scenarios in which only individuals ages 12 and over were vaccinated.

## 3. Results

### 3.1. Impact of school opening on COVID-19 hospitalizations

**Figure 2.**
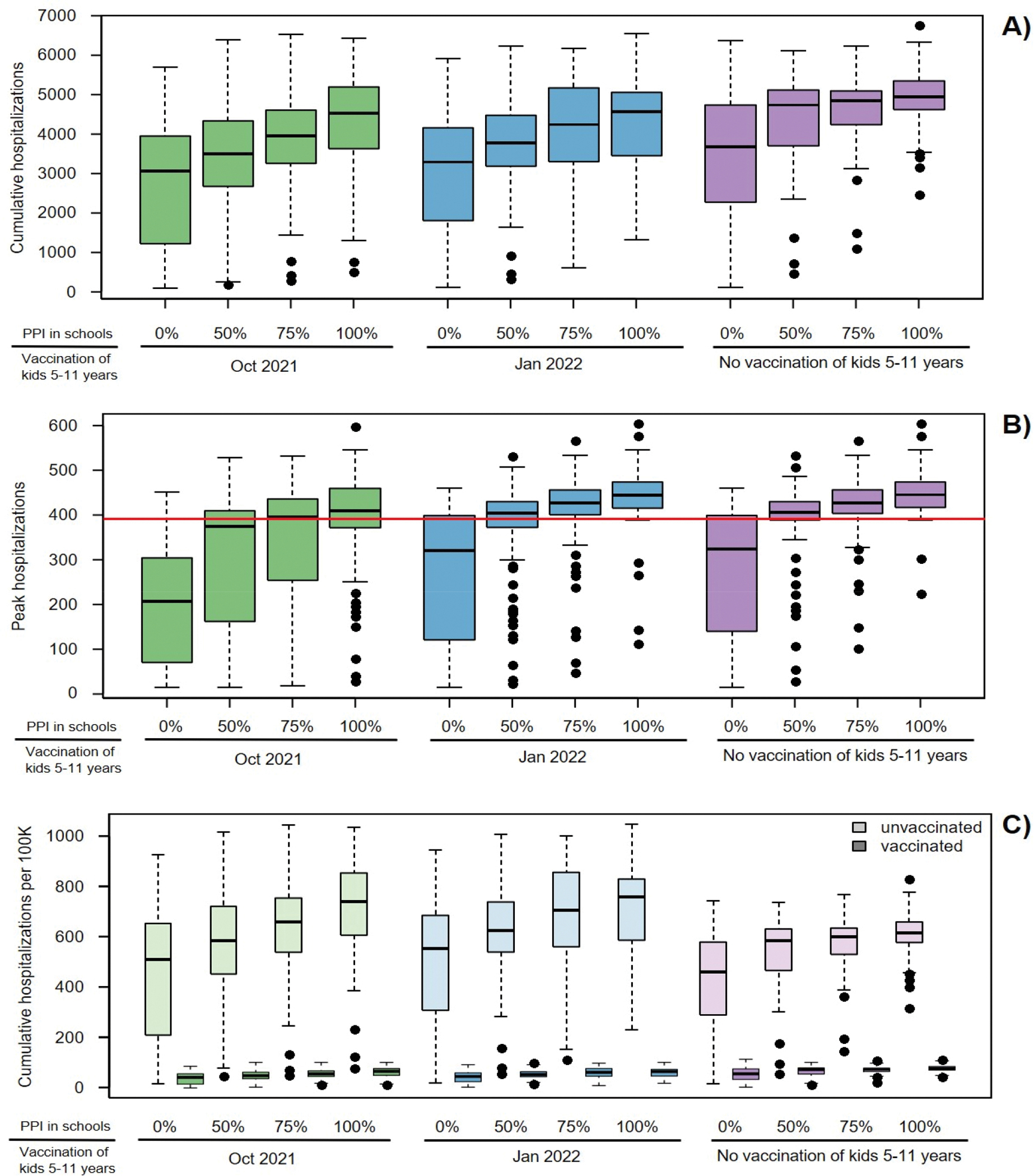
Overall hospitalizations expected under different scenarios of school reopening and extended vaccine eligibility: A) Cumulative hospitalizations over the 2021–2022 school year; B) The maximum number of people hospitalized with COVID-19 at any given time over the school year and C) Overall per capita cumulative hospitalizations by vaccination status. Boxes represent interquartile range while whiskers extend to the most extreme data point which is no more than 1.5 times the interquartile range. Red line indicates 10% of the hospital bed capacity in King County used as a metric for public-health decisions.

**Figure 3.**
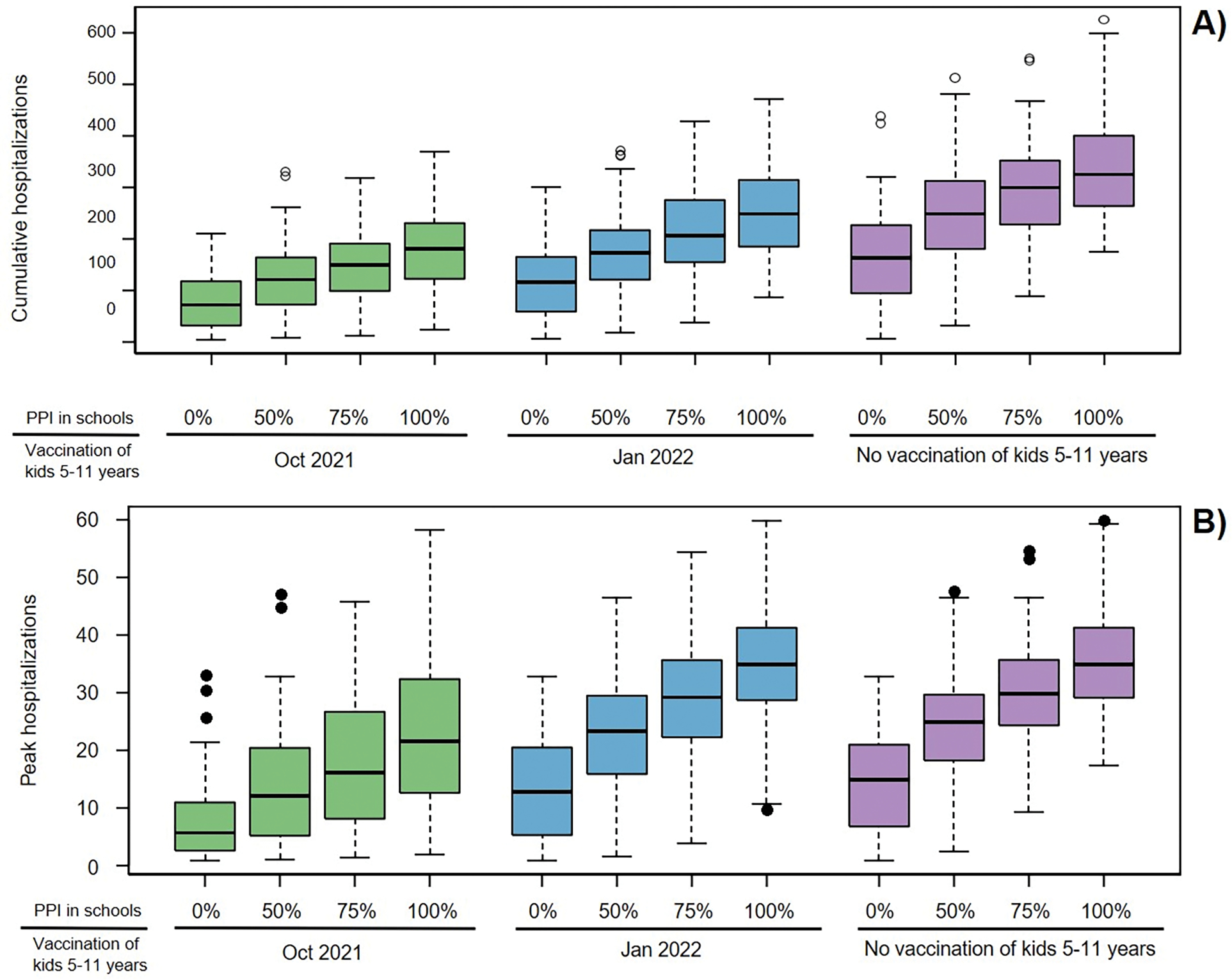
Hospitalizations among the youngest group (0–19 years) expected under different scenarios of school reopening and extended vaccine eligibility: A) Cumulative hospitalizations over the 2021–2022 school year and B) The maximum number of young people hospitalized with COVID-19 at any given time over the school year. Boxes represent interquartile range while whiskers extend to the most extreme data point which is no more than 1.5 times the interquartile range.

**Figure 4.**
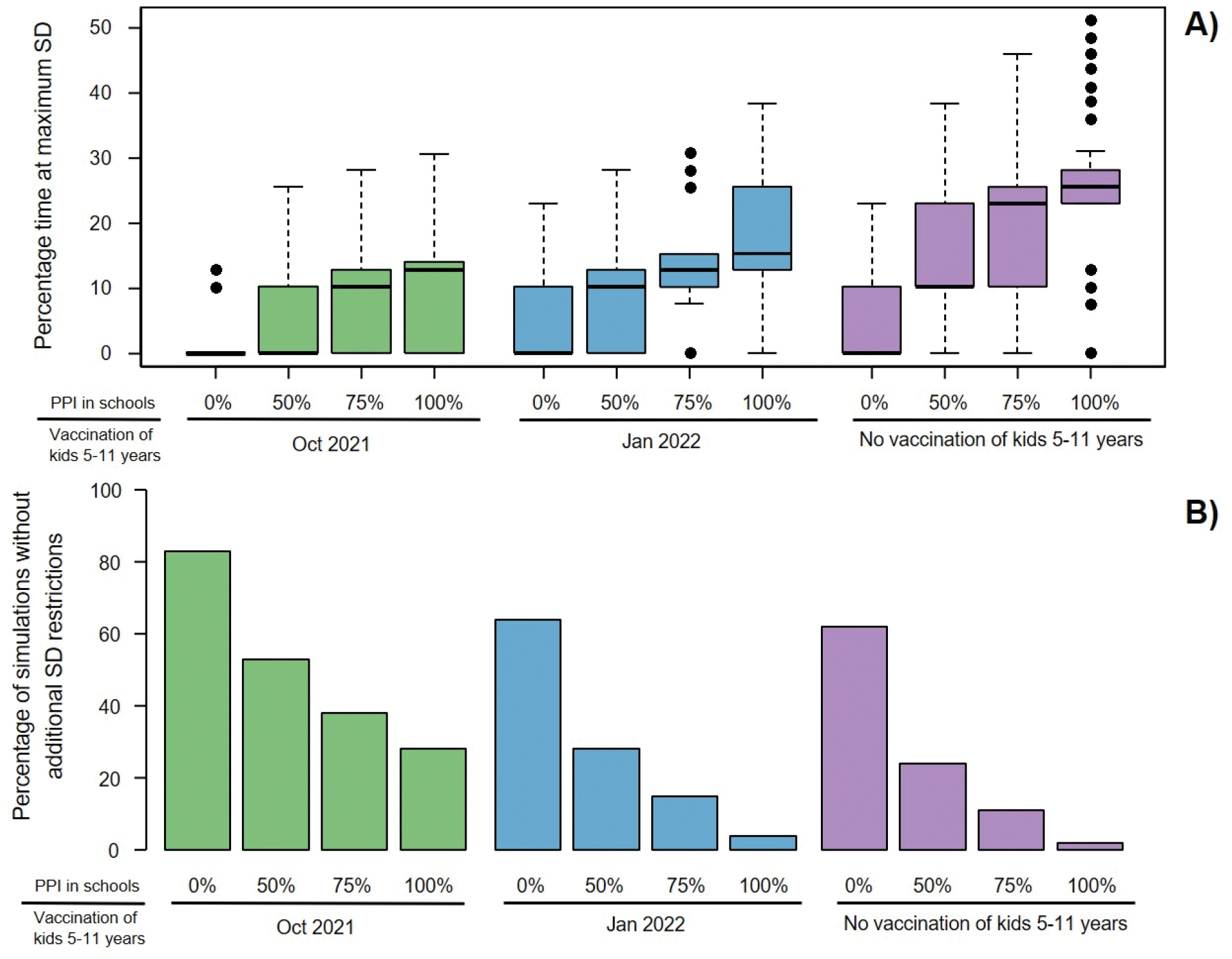
Effects of school reopening and extended vaccine eligibility on the need of additional COVID-19 restrictions. A) Percentage time of the school year under maximum restricted social distancing (SD) and B) Percentage of simulations in which additional restrictions of social distancing are not required. Boxes represent interquartile range while whiskers extend to the most extreme data point which is no more than 1.5 times the interquartile range.

Assuming 85% of the currently eligible population at the time of analysis (12 years and older) was vaccinated, implies that 72.9% of the total population was vaccinated with approximately 33% of the unvaccinated population being children of school age. In the absence of further vaccination our analysis showed that reopening schools in Fall 2021 will likely have a significant impact on expected SARS-CoV-2 transmission in King County resulting in more hospital admissions over the school year. Our model projected 4945 (median, IQR 4622–5341) total COVID-19 hospitalizations if physical contacts in schools were restored to their pre-COVID-19 level (100% PPI) for the entire school year with 445 (median, IQR 418–473) peak hospitalizations compared to 3675 (median, IQR 2311–4725) total and 324 (median, IQR 141–399) peak hospitalizations if schools remained closed (Figure 2A,B, purple). Impacts were even greater for projected pediatric hospitalizations (age 0–19), increasing from 163 to 325 (median, Figure 3A, purple). Reopening schools also impacted the projected amount of time when maximum social distancing is imposed to limit COVID-19 outbreak, increasing from a median of 0% (median, IQR 0–10) to 26% (median, IGQ 23–28) (Figure 4A, purple) and decreasing the fraction of simulations avoiding restrictions to 2% (Figure 4B, purple). Most hospitalizations were projected to occur among unvaccinated people (see Figure S7) at a per capita rate nearly 8-fold higher compared to vaccinated (median, Figure 2C)).

### 3.2. Impact of mitigation measures at schools

If children aged 5–11 were not vaccinated, reducing contacts in schools by 25 or 50% (through masking, ventilation and distancing) only slightly decreased the overall median cumulative hospitalizations (by 2 and 4% respectively, Figure 2) with more substantial effects on youth hospitalizations (median decreased by 8 and 23% respectively, Figure 3). Simulating 75 % PPI in schools of the pre-COVID-19 level shortened societal restrictions needed to keep COVID-19 hospitalization rates below state-mandated thresholds by one week and increases the proportion of simulations that avoid restrictions to 11 %. However, if physical contacts in schools were limited to 50 %, the duration of societal restriction was cut in half and the proportion of simulations avoiding restriction doubled to ~24 % (Figure 4). If reactive SD was not applied (results with fixed SD shown in Figure 6), the effect of school mitigation measures was much more apparent, with a 29 % reduction in overall hospitalization in the 50 % PPI vs 100 % PPI scenarios.

### 3.3. Impact of vaccinating children aged 5–11

Extending the vaccination eligibility to children aged 5–11 and achieving the same vaccination coverage (85%) in this age group would require approximately 150,000 additional vaccinations. It would increase the overall population vaccination coverage to 80.2% and reduce the proportion of unvaccinated who are school age to 11%. Between 400 and 1200 fewer hospitalizations were projected over the school year with vaccination of children 5–11 starting in October 2021 across scenarios with different levels of PPI in schools (Figure 2A, green). Regardless of the level of physical interactions in schools, the expected number of hospitalizations in the youngest age group was roughly cut in half (Figures 3A and S9B, green). Moreover, offering early vaccination to children aged 5–11 results in reducing the need of additional social distancing (Figure 4, green). For instance, in the scenario with 75% PPI in schools, the median number of hospitalizations over the school year was projected to be 13% lower than if school contacts were fully restored (100% PPI) with only slight reduction in the projected time under societal restrictions. However, controlling physical contacts in schools to 50% of the pre-COVID-19 level (50% PPI) reduced the projected median number of hospitalizations by an additional 23% and also increases the proportion of simulations avoiding societal restrictions above 50%. The projected proportion of hospital beds filled with COVID-19 patients also remained below the county target of 10% (Figure 2B, green) [36]. Delaying childhood vaccination by 3 months voided most of the benefits in terms of peak hospitalizations (Figures 2B and 3B, blue) over the school year but still prevented 25–30% of the pediatric hospitalizations and reduced time at maximal social distancing (Figures 4 and S9B, blue).

### 3.4. Impact of increasing overall vaccination coverage

**Figure 5.**
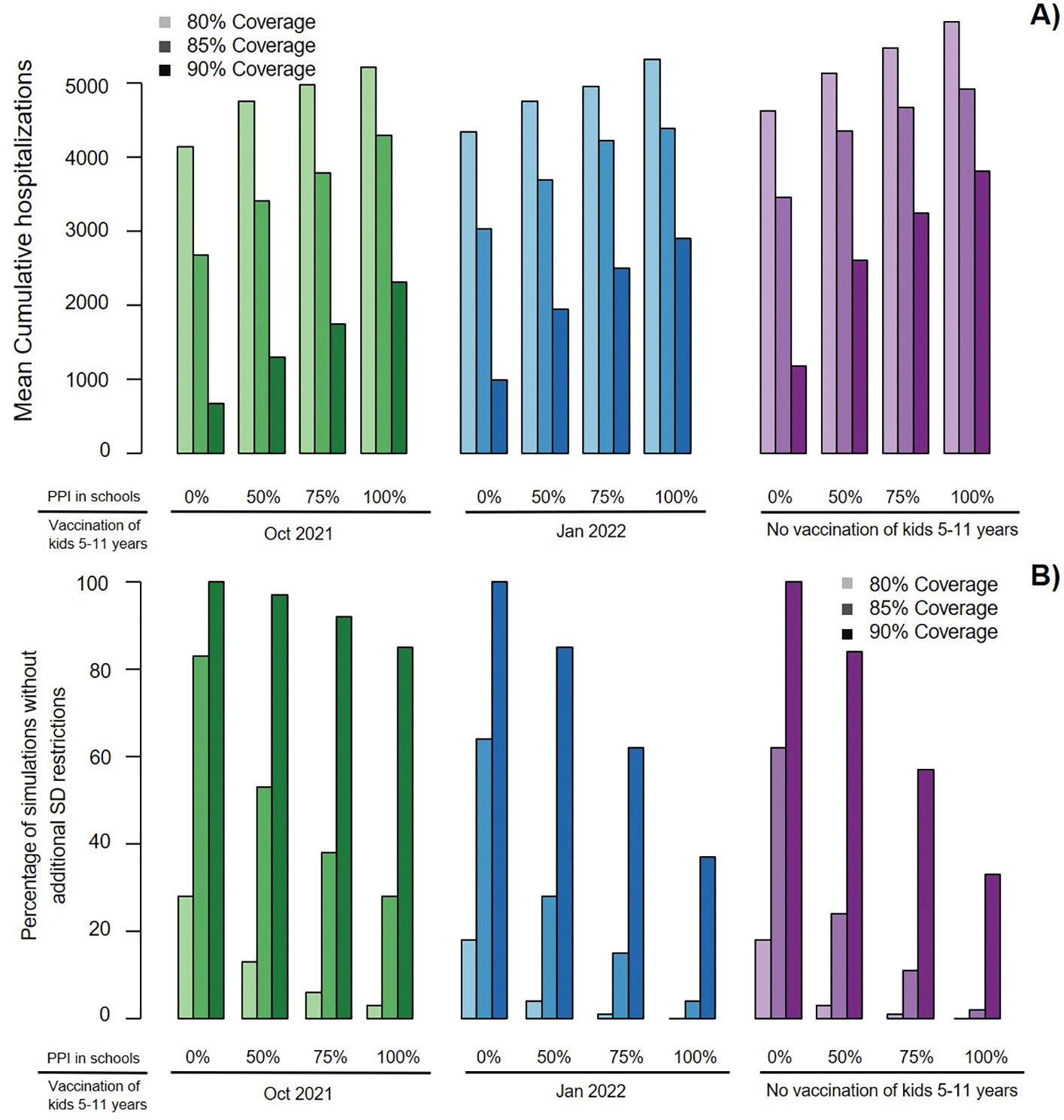
Importance of achieving better vaccination coverage. A) Mean cumulative hospitalizations over the school year and B) Percentage of simulations in which additional restrictions of social distancing are not required.

We explored the effects of improved vaccination coverage in King County. Increasing the proportion of vaccinated individuals aged 12+ to 90% required approximately 100,000 additional vaccinations and was projected to have a significant effect on the expected hospitalizations over the school year across all scenarios. If children aged 5–11 remained unvaccinated with 75% PPI in schools, reaching 90% overall vaccination coverage prevented on average approximately 1300 hospitalizations or one quarter of the projected (Figure 5A, purple) but still almost 50% of the simulations required additional social distancing (Figure 5B, purple). The benefit of vaccinating 90% of the eligible population was largest when combined with early vaccination of children aged 5–11. In those scenarios it was projected to prevent ~2400 hospitalizations on average over the school year which is 60% of those estimated with 85% coverage (Figure 5A, green) and avoided the need of extra periods of social distancing (Figure 5B, green). Conversely, if the overall vaccination coverage remained at the level reached in September 2021 (80%) we projected more hospitalizations across scenarios and a very slim chance to avoid additional societal restrictions.

### 3.5. Importance of reactive social distancing

**Figure 6.**
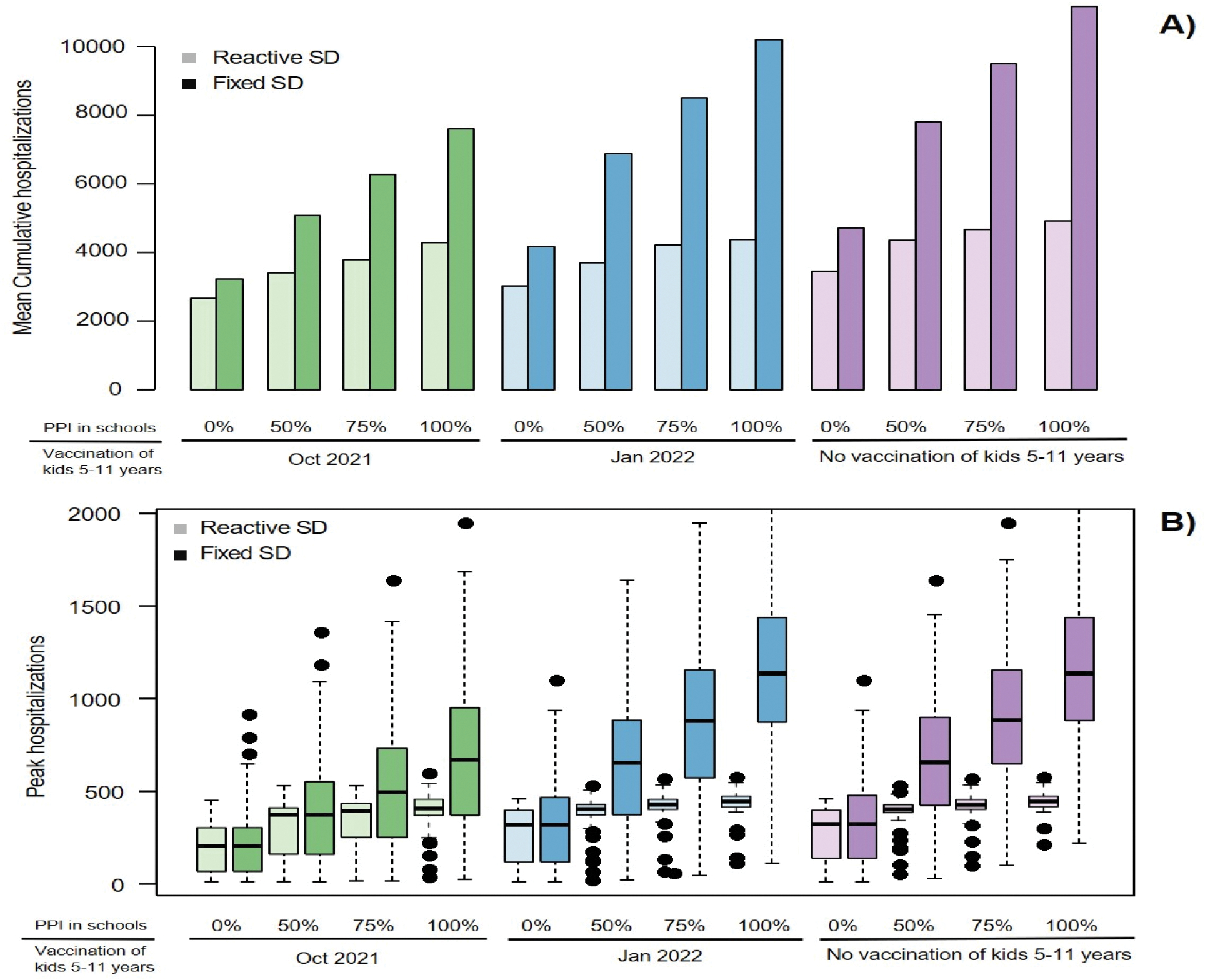
Importance of reactive social distancing. A) Mean cumulative hospitalizations and B) peak hospitalization over the school year under scenarios with reactive social distancing compared to scenarios in which restrictions are kept at the minimum level (*SDmin*). Boxes represent interquartile range while whiskers extend to the most extreme data point which is no more than 1.5 times the interquartile range.

Our rules of reactive social distancing created some simulations in which expanding vaccination resulted in significantly shorter time with additional restrictions but more hospitalizations over the school year (Figure S8). Alternative scenarios in which reductions in social interactions were kept at the lowest level (*SDmin*) throughout the school year avoided this outcome and always resulted in reduced number of hospitalizations (Figure S10). However, in such scenarios without reactive social distancing the number of hospitalizations over the school year was expected to surpass 10,000 (an increase by 123% compared to reactive social distancing) if vaccination was not offered to children aged 5–11 (Figure 6, purple). If all school-aged children were eligible for early vaccination the projected number of hospitalizations with 100% PPI in schools was approximately 8000 (65% increase) and could be kept below 5000 only if less than 50% PPI was maintained at schools (Figure 6A, green). Notably, all scenarios of in-person schooling resulted in more than 500 COVID-19 hospitalizations at a time without reactive social distancing (Figure 6B). Simulations with different ranges of social distancing (Figure S11) demonstrated that it is more important to keep sufficient level of minimum physical distancing (higher *SDmin*) than imposing stronger restrictions when hospitalizations increase (higher *SDmax*).

### 3.6. Simulations with immune waning

We also simulated scenarios in which protection against infection provided by vaccine or previous infection decreased by half over an average of a year while preserving protection against hospitalization (Figure S12). Our model projected: i) between 8000 and 10,000 hospitalizations under these scenarios, representing a 2- to 3-fold increase over projections from our main analysis; ii) large increases of the expected peak hospitalizations up to 650–750 and iii) additional social restrictions needed for more than 75% of the school year. These results suggest that more stringent restrictions of social interactions combined with mass vaccine boosting may be necessary.

## 4. Discussion

Our simulations suggest that due the highly infectious nature of the SARS-CoV-2 Delta variant, keeping schools open with contacts at pre-pandemic levels (no in-school masking, physical distancing or testing) would have resulted in unmanageable numbers of hospitalizations in King County in the fall of 2021, despite high overall vaccination rates. These results agree with previous work and have been largely validated by the current experiences across the United States [29,39,40]. States with low vaccination rates where all non-pharmaceutical interventions have been lifted witnessed the largest number of hospitalizations in recent months, putting their healthcare systems at the brink of collapse [38,41,42]. Our results align with other models suggesting that without school-based interventions, the majority of school-age students would become infected during the school year [29]. Lessler et al. showed that seven simultaneous mitigation strategies were needed at schools to significantly reduce school-associated risk [27]. Consistent with this finding, we showed that reducing potentially infectious contacts to 50% of pre-pandemic levels would result in a significant reduction of hospitalizations and infections in school-age children. We demonstrated that in the current environment of reactive social distancing, an effective intervention significantly reducing transmission may result in a comparable number of cases or hospitalizations over a relatively long term (9–12 months) but allow society to retain maximum level of physical interactions. Therefore, estimating the societal cost of the epidemic in terms of projected time at elevated social distancing should be considered in combination with the health metrics such as hospitalizations or deaths when the overall impact of a given intervention is evaluated.

Pediatric cases in the US increased exponentially in the first weeks of September 2021, and hospitalizations for children and adolescents increased 5-fold between June and mid-August with the rise of the Delta variant, prompting the American Academy of Pediatrics to plead with the FDA to accelerate vaccine approval for the pediatric population [43,44]. Pfizer announced favorable preliminary results for the clinical trials for their vaccine in children aged 5 to 11 [15]. We demonstrated that vaccination of this age group would further reduce the burden of COVID-19 disease in children, averting up to 60% hospitalizations in that age group.

Finally, we reiterated the importance of targeting the remaining unvaccinated population of adults for vaccination. Additional 5% vaccination coverage would result in a disproportionate reduction in hospitalizations and could eliminate the need for any periods of required further social distancing. Although seemingly small, such improvement may require promoting all best practices at the state level to be achieved, given that the vaccination coverage in King County is already high. However, data from countries like Ireland with 90.3% of the adult population vaccinated including close to 100% of seniors (65+) is encouraging [45].

As of November 14, 2021, 83.5% of King County residents over the age of 12 have completed their vaccine series with vaccination for children aged 5–11 just started. Schools remained open with mask mandates instituted indoors and at large outdoor events. There have been more that 1000 cumulative hospitalizations since September 1 reaching daily maximum of ~30. Overall, these results suggest that PPI were likely maintained at a relatively low level in schools, perhaps due to masking, improved ventilation, and distancing in classrooms. Moreover, vaccination in the population at large, as well as maintained use of NPIs in the community, was sufficient to prevent a wave large enough to require a large increase in social distancing.

As with any simulation-based analysis, our work has several limitations. We assumed vaccines protect vaccinated individuals immediately after vaccination, and we did not explicitly model two-dose vaccination. Our model is stratified by age, and we do not consider other factors such as occupation, socio-economic status and race that are known to affect both susceptibility to infection and to severe disease. Factors such as time-varying diagnostic rates, different levels of cross-immunity for re-infections with different strains and the multifactorial components of social distancing which cannot be measured or predicted may influence the estimated number of cases and hospitalizations. As a consequence, our model is not a forecasting tool and that not intended to make specific projections for other regions or counties where vaccination rates, masking, age structure, and SARS-CoV-2 seroprevalence differ. However, the model’s qualitative conclusions are likely to be highly relevant for most communities.

Overall, our work highlights three main messages. First, nonpharmaceutical interventions must be maintained vigilantly in schools to prevent both pediatric and adult hospitalizations. Second, vaccination of school-aged children will only exert a meaningful impact on the epidemic over this school year if implemented rapidly. We project that this will also be the case for boosting against new variants. Finally, it remains critical to vaccinate and boost as many unvaccinated adults as possible to limit the morbidity and mortality associated with newly emerging variants. Each of our conclusions become even more urgent when we consider the possibility of immune waning for previously infected and vaccinated people as well as the spread of more transmissible variants such as Omicron.

## Supplementary Material

Supplementary Materials

## Figures and Tables

**Table 1.** Key model parameters.

Parameter description	Value	Source

**Maximum level of social distancing** *(SDmax)* by age (younger/older than 70)	(0.3/0.5)	[28]
**Minimum level of social distancing** *(SDmin)* by age (younger/older than 70)	(0.1/0.2)	[46]
**Threshold for triggering additional restrictions** on social interactions	more than 10 new hospitalizations per 100,000 individuals per week	[36,47]
**Threshold for releasing restrictions** on social interactions	fewer than 5 new hospitalizations per 100,000 individuals per week	[36,47]
**Vaccination rate defined as number of people initiating series per day**	**5000**	**Assumed**
**Vaccine efficacy on susceptibility** defined as reduction of the probability to acquire infection upon exposure by variant (Alpha, Delta)	(0.91, 0.82)	[5,48]
**Vaccine efficacy on symptomatology** defined as reduction in the probability to develop symptoms upon infection by variant (Alpha, Delta)	(0.34, 0.34)	[5]
**Vaccine efficacy on hospitalization** defined as reduction in the risk of hospitalization for symptomatic infections by variant (Alpha, Delta)	(0.67, 0.67)	[49]
**Vaccine efficacy on infectiousness** defined as reduction in the probability to transmit different variants (Alpha, Delta) upon infection	(0, 0)	Assumed
**Relative transmissibility** of variants (Alpha, Delta) compared to the original variant	(1.5, 2.4)	[50–53]
**Relative severity** of variants (Alpha, Delta) compared to the original variant	(1.5, 1.5)	[4,54,55]
**Proportion of infections which become symptomatic** by age group (0–19, 20–49, 50–69, 70+)	(0.25, 0.33, 0.55, 0.70)	[56]
**Proportion of symptomatic infections which are mild** and require no hospitalizations by age group (0–19, 20–49, 50–69, 70+)	(0.988–0.996, 0.96–0.995, 0.87–0.96, 0.62–0.87)	Calibrated
**Relative susceptibility** by age group (younger/older than 20)	(0.5/1)	[56]

**Table 2.** Model parameters defining scenarios included in the main and sensitivity analyses (bold values are used in the main analysis).

Parameter/Description	Values

**Proportions of physical interactions (PPI)** in schools relative to pre-COVID-19 time	**0% (schools closed), 50%, 75%, 100%**
**Start of vaccinating children ages 5–11**	**Oct 1, 2021, Jan 1, 2022, never**
**Overall vaccination coverage** achieved among subpopulation eligible for vaccination	80%, **85%**, 90%
**Social distancing (SD) level**, defined as percentage reduction of COVID-19 transmission due to limiting physical interactions including state and local mandates, masking, remote work, etc.	• **Reactive: varied within ranges listed in** Table 1 **in response to recorded number of hospitalizations over time** • Fixed at minimum (10%)
**Waning of protection** against infection due to vaccination or prior infection	• **No waning** • Waning to 50% of initial efficacy on susceptibility over 1 year without effect on the efficacy on hospitalization
